# Lipopolysaccharide modulates astrocytic S100B secretion: a study in cerebrospinal fluid and astrocyte cultures from rats

**DOI:** 10.1186/1742-2094-8-128

**Published:** 2011-10-04

**Authors:** Maria Cristina Guerra, Lucas S Tortorelli, Fabiana Galland, Carollina Da Ré, Elisa Negri, Douglas S Engelke, Letícia Rodrigues, Marina C Leite, Carlos-Alberto Gonçalves

**Affiliations:** 1 Departamento de Bioquímica, Instituto de Ciências Básicas da Saúde, Universidade Federal do Rio Grande do Sul, Ramiro Barcelos, 2600- Anexo, 90035-003, Porto Alegre, Brazil

**Keywords:** astrocyte, GFAP, glutathione, LPS, TLR4, S100B

## Abstract

**Background:**

Inflammatory responses in brain are primarily mediated by microglia, but growing evidence suggests a crucial importance of astrocytes. S100B, a calcium-binding protein secreted by astrocytes, has properties of a neurotrophic or an inflammatory cytokine. However, it is not known whether primary signals occurring during induction of an inflammatory response (e.g. lipopolysaccharide, LPS) directly modulate S100B.

**Methods:**

In this work, we evaluated whether S100B levels in cerebrospinal fluid (CSF) and serum of Wistar rats are affected by LPS administered by intraperitoneal (IP) or intracerebroventricular (ICV) injection, as well as whether primary astrocyte cultures respond directly to lipopolysaccharide.

**Results:**

Our data suggest that S100B secretion in brain tissue is stimulated rapidly and persistently (for at least 24 h) by ICV LPS administration. This increase in CSF S100B was transient when LPS was IP administered. In contrast to these S100B results, we observed an increase in in TNFα levels in serum, but not in CSF, after IP administration of LPS. In isolated astrocytes and in acute hippocampal slices, we observed a direct stimulation of S100B secretion by LPS at a concentration of 10 μg/mL. An involvement of TLR4 was confirmed by use of specific inhibitors. However, lower levels of LPS in astrocyte cultures were able to induce a decrease in S100B secretion after 24 h, without significant change in intracellular content of S100B. In addition, after 24 h exposure to LPS, we observed a decrease in astrocytic glutathione and an increase in astrocytic glial fibrillary acidic protein.

**Conclusions:**

Together, these data contribute to the understanding of the effects of LPS on astrocytes, particularly on S100B secretion, and help us to interpret cerebrospinal fluid and serum changes for this protein in neuroinflammatory diseases. Moreover, non-brain S100B-expressing tissues may be differentially regulated, since LPS administration did not lead to increased serum levels of S100B.

## Background

S100B is a small very soluble calcium-binding protein that is highly expressed and secreted by astrocytes in the central nervous system (see [1] for a review). This protein has many putative intracellular targets (e.g. glial fibrillary acidic protein, GFAP) and, like other protein members of the S100 family, is involved in regulation of the cytoskeleton and the cell cycle. Moreover, extracellular S100B at nanomolar levels in *in vitro *assays has trophic effects on astrocytes, neurons and microglia. Many modulators of S100B secretion have been described in astrocyte preparations, such as forskolin, lyso-phosphatidic acid [2], fluoxetin [3] and kainate [4]. S100B secretion is also affected by metabolic stress conditions such as elevated concentrations of glutamate [5], glucose [6] and ammonium [7]. Other cells in the brain (e.g. oligodendrocytes [8]) and outside (e.g. adipocytes [9]) also express this protein, but whether S100B is secreted by these cells and which secretagogues are involved remain to be better characterized.

S100B has been proposed as a marker of astroglial activation in brain disorders, and changes in its cerebrospinal fluid and/or serum content have been associated with various neurological and psychiatric diseases [10,11]. Such disorders commonly have an important inflammatory component, in which S100B has often been thought of as a cytokine. Recently, we demonstrated that IL-1β modulates S100B secretion in astrocyte cultures and hippocampal slices [12]. Moreover there is evidence that S100B modulates and is modulated by pro-inflammatory cytokines [13-15]. However, we do not know if primary signals in the induction of inflammatory responses (e.g. LPS) directly modulate S100B.

Astrocytes are the most abundant glial cells in the brain, where they play key roles in neurotransmitter metabolism, antioxidant defense and regulation of extracellular concentration of potassium [16]. GFAP, as mentioned above, is a specific marker of astrocytes and, frequently, its elevation is a strong sign of astrogliosis, which occurs in several conditions involving brain injury [17].

LPS, a component of the cell wall of gram-negative bacteria, has been widely used experimentally to stimulate inflammatory responses, including in the central nervous system (e.g. [18]). Inflammatory response in the brain is primarily mediated by microglia, but growing evidence suggests a crucial importance of astrocytes as well [19]. Like microglia, these cells have a toll-like receptor type 4 (TLR4), which belongs to TLR family receptors in the vertebrate immune system and specifically recognizes LPS [20].

Recent studies have shown that astrocytes respond to LPS, decreasing expression of proteins such as gap junction proteins [21], and increasing expression of others such as GFAP and glutathione-S-transferase [22,23]. Interestingly, we have demonstrated that gap junction inhibitors increase secretion of S100B from astrocytes and hippocampal slices [24].

Our working hypothesis was that S100B is released by astrocytes as a cytokine in response to LPS. In this study, we evaluated whether S100B content in cerebrospinal fluid (CSF) and serum of rats is affected by LPS administered by intraperitoneal or intracerebroventricular injection, as well as whether astrocyte cultures and acute hippocampal slices respond directly to LPS. In parallel, we investigated whether LPS affects the content of GFAP and glutathione in astrocyte cultures, as indices of astrogliosis (GFAP) and antioxidant defense (based on capacity for synthesis and release of glutathione). Moreover, we measured the profile of secretion of TNFα, a cytokine that is well-known to respond to LPS.

## Methods

### Materials

Poly-L-lysine, antibody anti-S100B (SH-B1), methylthiazolyldiphenyl-tetrazolium bromide (MTT), neutral red, and lipopolysaccharides from *Escherichia coli *(LPS) 055:B5 were purchased from Sigma [St. Louis, USA]. Fetal calf serum (FCS), Dulbecco's modified Eagle's medium (DMEM) and other materials for cell culture were purchased from Gibco [Carlsbad, USA]. Polyclonal anti-S100B and anti-rabbit peroxidase linked were purchased from DAKO [São Paulo, Brazil] and GE [Little Chalfont United Kingdom], respectively. Inhibitors for TLR4 (CLI-095 and OxPAPC) were from InVivoGen [San Diego, USA].

### Surgical procedure for intracerebroventricular (ICV) LPS infusion

Procedures were carried out in accordance with the NIH Guide for the Care and Use of Laboratory Animals and were approved by the local authorities. Adult Wistar rats (90 days old) were used. For ventricular access, the animals were anesthetized with ketamine/xylazine (75 and 10 mg/Kg, respectively, i.p.) and placed in a stereotaxic apparatus. A midline saggital incision was made in the scalp and one burr hole was drilled in the skull over both ventricles. The following coordinates were used: 0.9 mm posterior to bregma; 1.5 mm lateral to saggital suture; 3.6 mm beneath the brain surface [25]. The rats received 5 μL ICV/side of LPS 2.5 ug/μL or phosphate-buffered saline (control). After the surgical procedure, rats were kept in a stereotactic holder for 30 min or 24 h and CSF was obtained by puncture of the cisterna magna using an insulin syringe. A maximum volume of 30 μL was collected over a 3-min period to minimize risk of brain stem damage. The blood samples were collected by careful intracardiac puncture, using a 5-mL non-heparinized syringe to obtain 3 mL of blood. Blood samples were incubated at room temperature (25°C) for 5 min and centrifuged at 3200 rpm for 5 min to obtain serum. Cerebrospinal fluid and serum samples were frozen (-70°C) until used for S100B or TNFα analysis.

### Intraperitoneal (IP) LPS infusion

Wistar rats (90 days old) were used for intraperitoneal injection of 0.3 mL of LPS, 250 μg/Kg, or phosphate-buffered saline (control). After 30 min or 24 h, the animals were anesthetized with ketamine/xylazine (75 and 10 mg/Kg, respectively, i.p.) and placed in a stereotaxic apparatus for CSF puncture. Blood samples were obtained by intracardiac puncture, and the animals were killed by decapitation.

### Cell culture

Primary astrocyte cultures from Wistar rats were prepared as previously described [26]. Procedures were carried out in accordance with the NIH Guide for the Care and Use of Laboratory Animals and were approved by the local authorities. Briefly, cerebral cortices of newborn Wistar rats (1-2 days old) were removed and mechanically dissociated in Ca^2+^- and Mg^2+^-free balanced salt solution, pH 7.4, containing (in mM): 137 NaCl; 5.36 KCl; 0.27 Na_2_HPO_4_; 1.1 KH_2_PO_4 _and 6.1 glucose. The cortices were cleaned of meninges and mechanically dissociated by sequential passage through a Pasteur pipette. After centrifugation at 1400 RPM for 5 min the pellet was resuspended in DMEM (pH 7.6) supplemented with 8.39 mM HEPES, 23.8 mM NaHCO_3_, 0.1% amphotericin, 0.032% gentamicin and 10% fetal calf serum (FCS). Cultures were maintained in DMEM containing 10% FCS in 5% CO_2_/95% air at 37°C, allowed to grow to confluence, and used at 15 days in vitro.

### Hippocampal slices

Hippocampal slices were prepared as previously described [27]. Procedures were carried out in accordance with the NIH Guide for the Care and Use of Laboratory Animals and were approved by the local authorities. Thirty-day old Wistar rats were killed by decapitation and the brains were removed and placed in cold saline medium with the following composition (in mM): 120 NaCl; 2 KCl; 1 CaCl_2_; 1 MgSO_4_; 25 HEPES; 1 KH_2_PO_4_, and 10 glucose, adjusted to pH 7.4 and previously aerated with O_2_. The hippocampi were dissected and transverse slices of 0.3 mm were obtained using a McIlwain Tissue Chopper. Slices were then transferred immediately into 24-well culture plates, each well containing 0.3 ml of physiological medium and only one slice. The medium was changed every 15 min with fresh saline medium at room temperature (maintained at 25°C). Following a 120-min equilibration period, the medium was removed and replaced with physiological saline with or without LPS for 60 min at 30°C on a warm plate. Afterwards, media were collected and stored at -70°C until used for assay of S100B or TNFα.

### S100B measurement

S100B was measured by ELISA, as previously described [28]. Briefly, 50 μl of sample plus 50 μl of Tris buffer were incubated for 2 h on a microtiter plate previously coated with monoclonal anti-S100B. Polyclonal anti-S100 was incubated for 30 min and then peroxidase-conjugated anti-rabbit antibody was added for a further 30 min. Color reaction with *o*-phenylenediamine was measured at 492 nm. The standard S100B curve ranged from 0.002 to 1 ng/ml.

### GFAP measurement

ELISA for GFAP was carried out, as previously described [29], by coating microtiter plates with 100 μL samples for 24 h at 4°C. Incubation with a polyclonal anti-GFAP from rabbit for 1 h was followed by incubation with a secondary antibody conjugated with peroxidase for 1 h, at room temperature. A colorimetric reaction with *o*-phenylenediamine was measured at 492 nm. The standard human GFAP (from Calbiochem) curve ranged from 0.1 to 5 ng/mL.

### MTT reduction assay

Cells were treated with 50 μg/mL Methylthiazolyldiphenyl-tetrazolium bromide (MTT) for 30 min in 5% CO_2_/95% air at 37°C. Afterwards, the media was removed and MTT crystals were dissolved in DMSO. Absorbance values were measured at 560 and 650 nm. The reduction of MTT was calculated as (absorbance at 560 nm) - (absorbance at 650 nm).

### Neutral red uptake

Neutral red incorporation was carried out as previously described [24] with modifications. Cells were treated with 50 μg/mL neutral red (NR) for 30 min in 5% CO_2_/95% air at 37°C. Afterwards, the cells were rinsed twice with PBS for 5 min each and NR dye taken up by viable cells was extracted with 500 μL of acetic acid/ethanol/water (1/50/49). Absorbance values were measured at 560 nm.

### Lactate dehydrogenase (LDH) assay

Lactate dehydrogenase assay was carried out in 50 μL of extracellular medium, using a commercial colorimetric assay from Doles (Goiânia, Brazil).

### Glutathione content

Glutathione content was determined as previously described [30]. Briefly, hippocampal slices or astrocyte cultures were homogenized in sodium phosphate buffer (0.1 M, pH 8.0) containing 5 mM EDTA and protein was precipitated with 1.7% meta-phosphoric acid. Supernatant was assayed with *o*-phthaldialdehyde (1 mg/mL of methanol) at room temperature for 15 min. Fluorescence was measured using excitation and emission wavelengths of 350 and 420 nm, respectively. A calibration curve was performed with standard glutathione solutions (0-500 *μ*M).

### Tumor necrosis factor α (TNFα) measurement

This assay was carried out in 100 μL of CSF, serum or extracellular medium, using a rat TNFα ELISA from eBioscience (San Diego, USA).

### Statistical analysis

Parametric data are reported as mean ± standard error and were analyzed by Student's *t *test (when two groups were considered) or by one-way analysis of variance (ANOVA) followed by Duncan's test, in the SPSS-16.0. Data from GFAP, S100B and TNFα measurements were log-transformed to satisfy the assumption of the statistical tests when necessary. Tests are specified in the legends, with level of significance set at p < 0.05.

## Results

### LPS induces increases in S100B levels in cerebrospinal fluid, but not in serum

**Figure 1:**
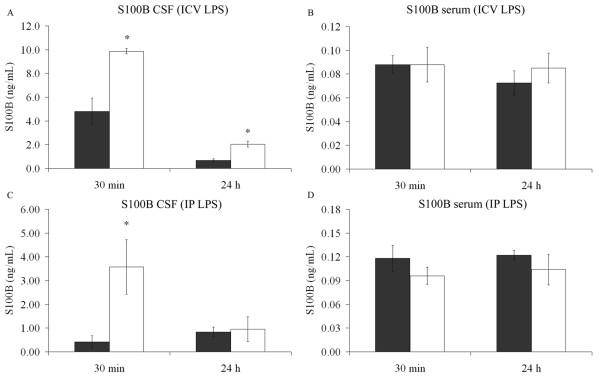
**LPS induces increased levels of S100B in cerebrospinal fluid (CSF), but not in serum**. Intracerebroventricular injection of LPS, or saline solution, was carried out in adult Wistar rats under anaesthesia. After 30 min or 24 h, cerebrospinal fluid was collected by magna puncture (A) and blood by intracardic puncture (B). The control group is represented by grey bars and the LPS-treated group is represented by open bars. Each value is a mean (± standard error) from 5 rats per group. Intraperitoneal infusion of LPS, or saline solution, was carried out in adult Wistar rats under anaesthesia. After 30 min or 24 h, CSF was collected by magna puncture (C) and blood by intracardic puncture (D). The control group is represented by grey bars and the LPS-treated group is represented by open bars. Each value is a mean (± standard error) from 5 rats per group. * Significantly different from respective control (Student t test, p < 0.05).

Anesthetized adult rats received 10 μL ICV of 2.5 μg/μL LPS or phosphate-buffered saline (control). CSF and blood were collected at 30 min or 24 h after LPS administration. A significant increase in CSF S100B was observed at 30 min (p = 0.009) and 24 h (p = 0.003) (Figure 1A), without significant changes in S100B serum content (p = 0.99, 30 min and p = 0.47, 24 h) (Figure 1B). Interestingly, when rats received IP LPS (250 μg/Kg body) they also exhibited an increase in CSF S100B at 30 min (p = 0.007), but not at 24 h (p = 0.68) (Figure 1C), and again no significant changes in serum S100B were observed when compared with controls that received phosphate-buffered saline (p = 0.28, 30 min and p = 0.32, 24 h) (Figure 1D). Notice that, assuming a mean body weight of rats of 0.3 Kg, the amount of LPS administered IP and ICV was 75 and 25 μg, respectively.

### LPS directly affects astrocytic S100B secretion, apparently without changing the intracellular content of this protein

**Figure 2:**
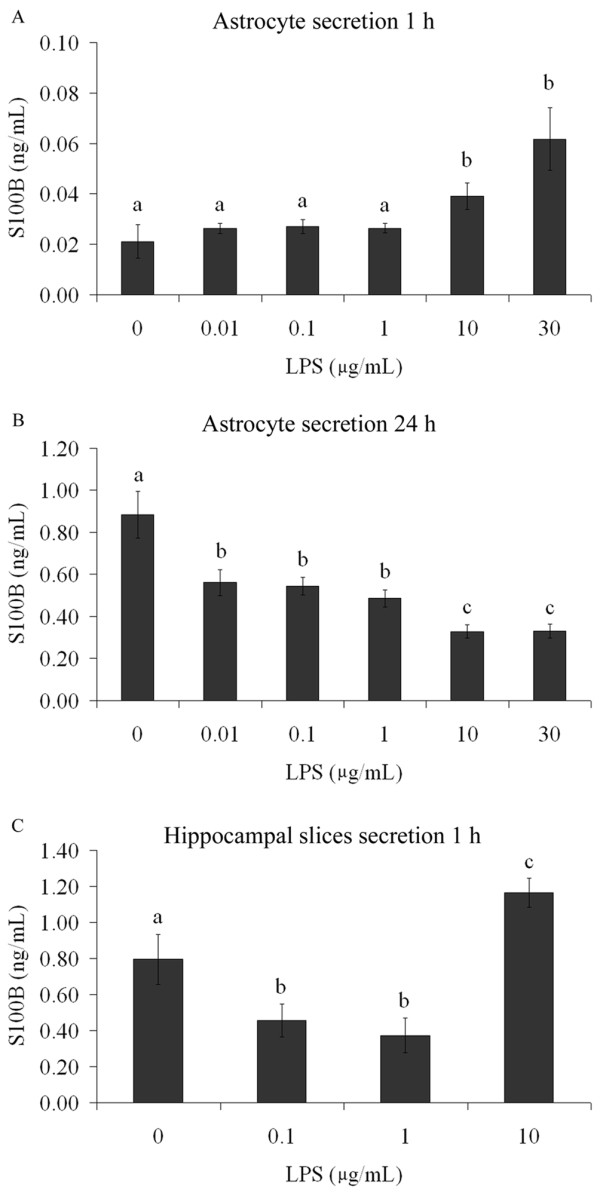
**S100B secretion is modified by LPS in astrocyte cultures and acute hippocampal slices**. Rat cortical astrocytes were cultured in DMEM containing 10% FCS. After confluence, the medium was replaced by DMEM without serum in the presence or absence of LPS (from 0.01 to 30 μg/mL). S100B was measured by ELISA at 1 h (A) and 24 h (B). Each value is a mean (± standard error) of at least 5 independent experiments performed in triplicate. Means indicated by different letters are significantly different, assuming p < 0.05. (C) Adult Wistar rats were killed by decapitation and 0.3 mm hippocampal slices were obtained using a McIlwain chopper. After a metabolic recovery period, hippocampal slices were exposed to LPS (from 0.1 to 10 μg/mL) and the extracellular content of S100B measured by ELISA at 1 h. Each value is the mean (± standard error) of at least 5 independent experiments performed in triplicate. Means indicated by different letters are significantly different (one way ANOVA followed by Duncan's test, with a significance level of p < 0.05).

**Figure 3:**
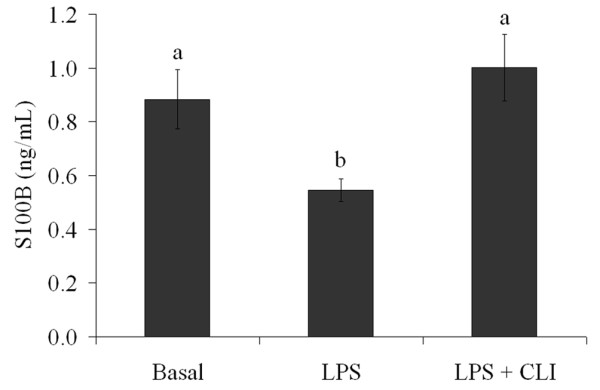
**The LPS-induced decrease in S100B secretion is abolished by inhibition of TLR4**. Rat cortical astrocytes were cultured in DMEM containing 10% FCS. After confluence, the medium was replaced by DMEM without serum in the presence or absence of 0.1 μg/mL LPS and 1 μM CLI-095, an inhibitor of TLR4. S100B was measured by ELISA at 24 h. Each value is a mean (± standard error) of at least 5 independent experiments performed in triplicate. Means indicated by different letters are significantly different (one way ANOVA followed by Duncan's test, with a significance level of p < 0.05).

In order to investigate whether this effect was attributable to a direct effect of LPS on astrocytes, we added different concentrations of LPS (from 0.01 to 30 μg/mL) to primary astrocyte cultures and extracellular S100B was measured at 1 h (Figure 2A) and 24 h (Figure 2B). At 1 h, LPS (at concentrations from 10 μg/mL upwards) increased S100B secretion (p < 0.001, ANOVA). Conversely, at 24 h, LPS caused a decrease in S100B secretion, even with LPS concentrations as low as 0.01 μg/mL (p < 0.001). Acute hippocampal slices were also exposed to LPS for 1 h (Figure 2C) and a decrease in S100B secretion was observed at LPS concentrations from 0.1 to 1 μg/mL (p < 0.001). However, LPS at 10 μg/mL produced an increase in S100B secretion (p < 0.001). In order to characterize whether the effect of LPS is mediated by TLR4, we incubated astrocytes with specific inhibitors for this receptor (Cli-095 and OxPAPC, at 1 μM and 30 μg/mL, respectively). Both CLI-095 (Figure 3) and OxPAPC (data not shown) abolished the effect of LPS. It is important to mention that OxPAPC *per se *increased S100B secretion and therefore it is difficult to affirm that this inhibitor prevented the effect induced by LPS.

**Figure 4:**
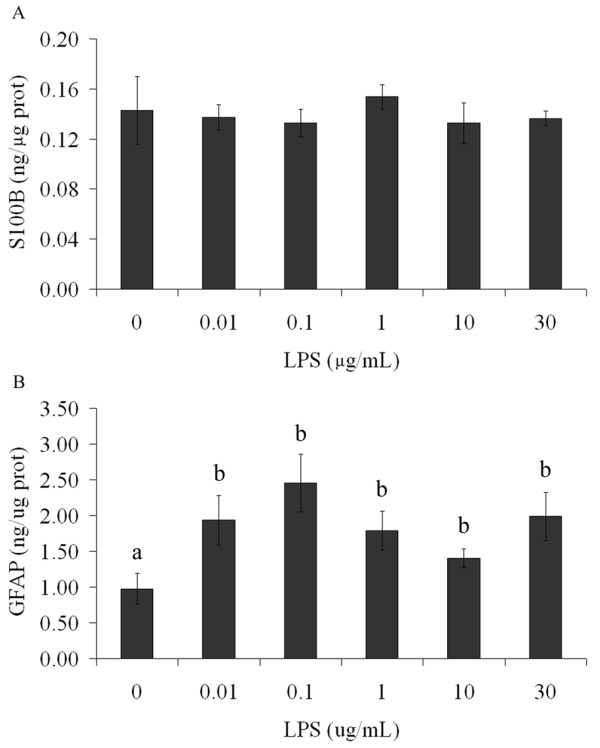
**Intracellular GFAP content is modified by LPS without change in intracellular S100B content in astrocytes**. Rat cortical astrocytes were cultured in DMEM containing 10% FCS. After confluence, the medium was replaced by DMEM without serum in the presence or absence of LPS (from 0.01 to 30 μg/mL). Cells were lysed and intracellular contents of S100B (A) and GFAP (B) were measured by ELISA. Each value is the mean (± standard error) of at least 5 independent experiments performed in triplicate. Means indicated by different letters are significantly different (one way ANOVA followed by Duncan's test, with a significance level of p < 0.05).

After 24 h of exposure to LPS, we measured S100B and GFAP content in lysed preparations of astrocyte cultures (Figure 4A and 4B, respectively). No significant changes were observed in S100B content (p = 0.85), but interestingly an increase in GFAP content was observed at all concentrations of LPS (p = 0.04).

### LPS decreases glutathione content, but does not affect cell viability and integrity

**Figure 5:**
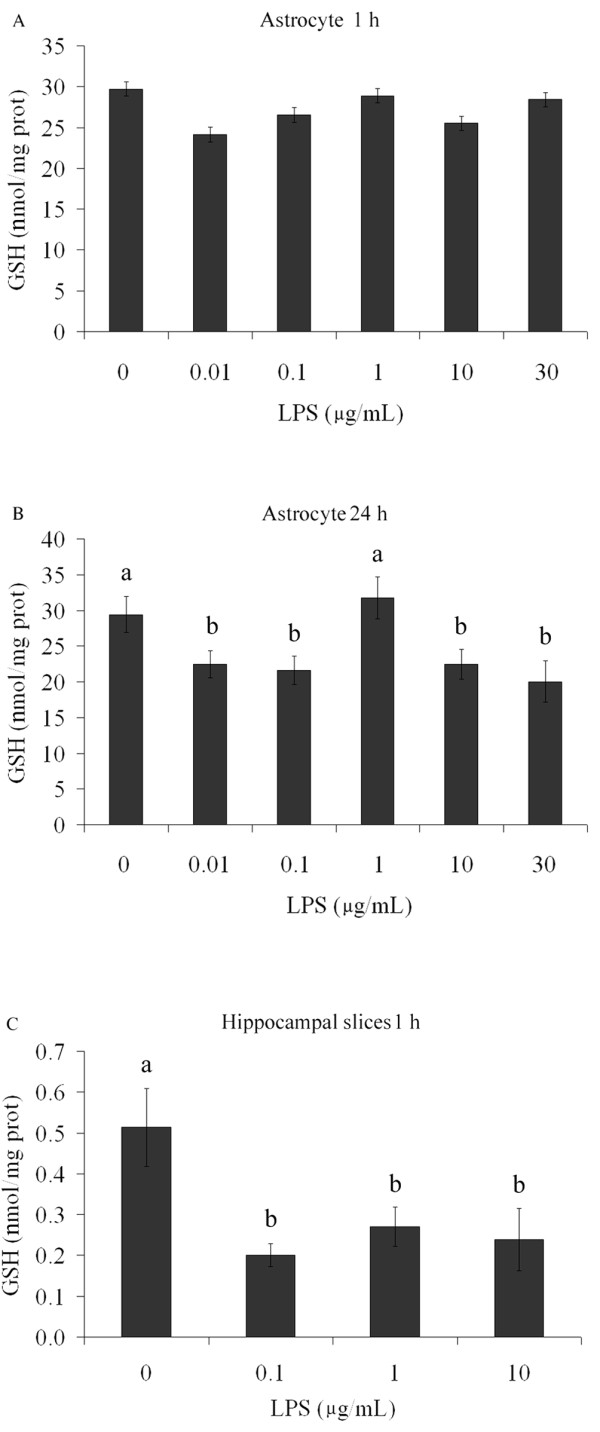
**GSH content is modified by LPS in astrocyte cultures and hippocampal slices**. Rat cortical astrocytes were cultured in DMEM containing 10% FCS. After confluence, the medium was replaced by DMEM without serum in the presence or absence of LPS (from 0.01 to 30 μg/mL). Cells were lysed in 1 h (A) or 24 h (B) and intracellular GSH content was measured. Each value represents the mean (± standard error) of at least 5 independent experiments performed in triplicate. Means indicated by different letters are significantly different (one way ANOVA followed by Duncan's test, with a significance level of p < 0.05). (C) Adult Wistar rats were killed by decapitation and 0.3 mm hippocampal slices were obtained using a McIlwain chopper. After a metabolic recovery period, hippocampal slices were exposed to LPS (from 0.1 to 10 μg/mL) and intracellular content of S100B was measured by ELISA at 1 h. Each value is the mean (± standard error) of at least 5 independent experiments performed in triplicate. Means indicated by different letters are significantly different (one way ANOVA followed by Duncan's test, with a significance level of p < 0.05).

Another parameter analyzed to evaluate astroglial activity was intracellular content of glutathione. After exposure of astrocytes to LPS (at concentrations from 0.01 to 30 μg/mL), we observed a decrease in intracellular content of glutathione after 24 h (p = 0.011), but not at 1 h (p = 0.49) (Figure 5A and 5B). Hippocampal slice preparations also exhibited a decrease in glutathione content after LPS exposure for 1 h (p = 0.015) (Figure 5C).

**Figure 6:**
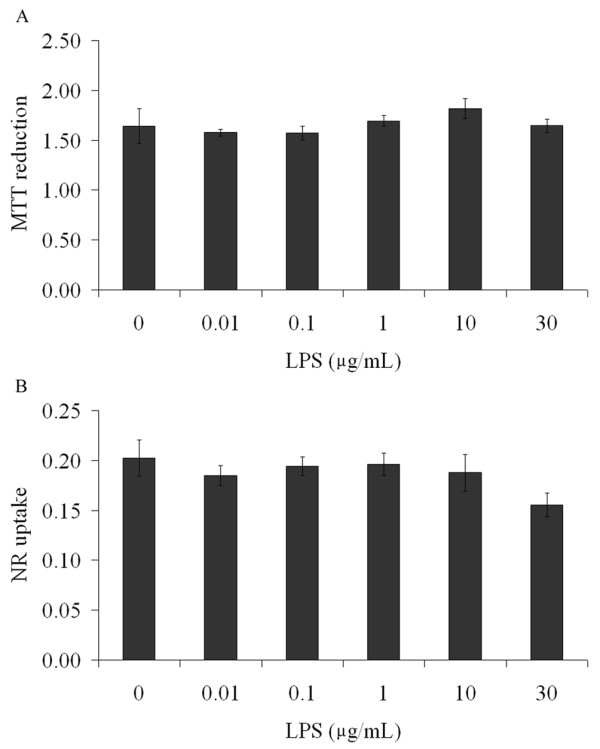
**LPS does not affect cell viability**. Rat cortical astrocytes were cultured in DMEM containing 10% FCS. Confluent astrocytes were exposed to LPS (from 0.01 to 30 μg/mL), during 24 h. At the end, cells were incubated with MTT (A) or neutral red (B). Each value is the mean (± standard error) of at least 5 independent experiments performed in triplicate. Statistical analysis was performed by one way ANOVA.

In order to detect a possible toxic effect of LPS in our preparations, we evaluated their capacities for MTT reduction, neutral red incorporation and LDH release. No changes in MTT reduction assay (p = 0.25) (Figure 6A) or in neutral red assay (p = 0.37) (Figure 6B) were induced in astrocyte cultures exposed to LPS (from 0.01 to 30 μg/mL). In addition, no changes in LDH release were seen (data not shown). Similar assays were also carried out in slice preparations confirming cell viability and integrity (data not shown).

### LPS induces an increase in TNFα in serum, but not in CSF

**Figure 7:**
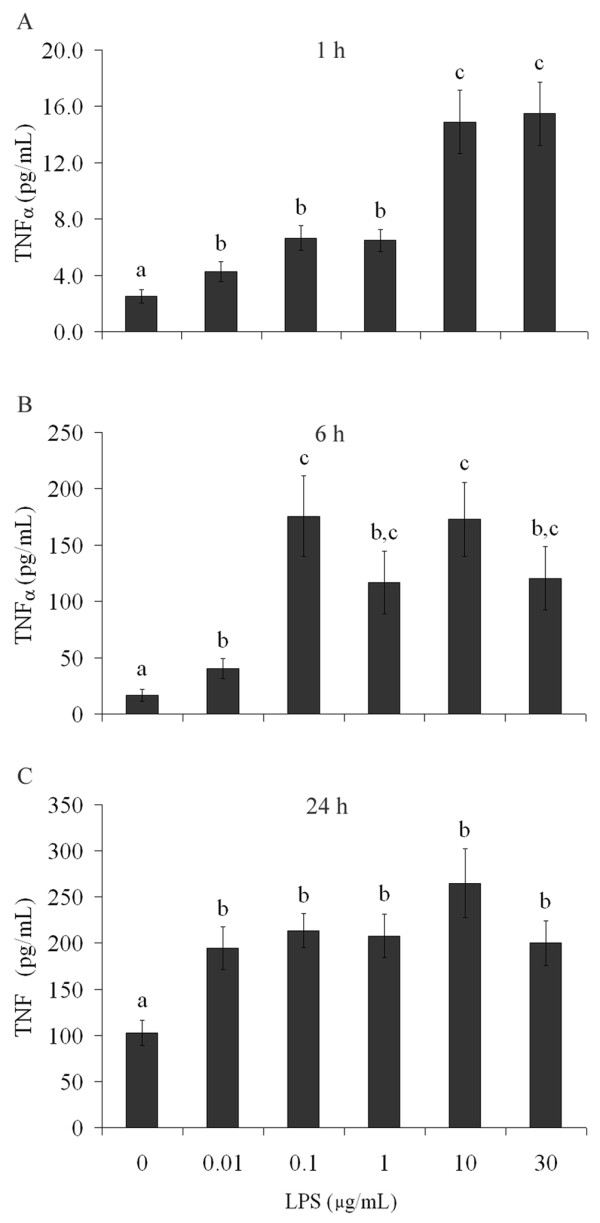
**TNFα secretion is modified by LPS in astrocyte cultures**. Rat cortical astrocytes were cultured in DMEM containing 10% FCS. After confluence, the medium was replaced by DMEM without serum in the presence or absence of LPS (from 0.01 to 30 μg/mL). TNFα was measured by ELISA at 1 h (A) and 6 h (B). Each value is a mean (± standard error) of at least 5 independent experiments performed in triplicate. Means indicated by different letters are significantly different (one way ANOVA followed by Duncan's test, with a significance level of p < 0.05).

Finally, we measured the response of the classic inflammatory cytokine, TNFα, to LPS *in vivo *to confirm the activity of this compound and to compare this response to that of S100B protein. In contrast to results for S100B, at 30 min and 24 h after IP administration of LPS (approximately 75 μg) we observed an increase in TNFα in serum (p = 0.04, 30 min and p = 0.04, 24 h), but not in CSF (p = 0.15, 30 min and p = 0.34, 24 h) (Table 1). When LPS (25 μg) was administered ICV we found an early and transient increase in TNFα in serum (p < 0.001) (at 30 min) and a later increase in CSF (p = 0.006) (at 24 h) (Table 2). In addition, we observed an increase in LPS-induced TNFα release from astrocyte cultures at 1, 6 and 24 h after exposure to LPS (Figure 7, p < 0.001). We were not able to detect TNFα release in acute hippocampal slices.

**Table 1:** Serum and CSF TNF_α _levels after IP administration of LPS in rats

	Control	LPS^a^	P
Serum (30 min)	3.4 ± 1.0	192.1 ± 97.2	0.046*
Serum (24 h)	1.1 ± 0.4	2.6 ± 0.2	0.021*
CSF (30 min)	2.7 ± 1.0	1.1 ± 0.5	0.145
CSF (24 h)	8.3 ± 5.5	2.5 ± 1.0	0.34

Values are mean (pg/mL) ± standard error (n = 5). Statistical analysis was performed using Student's *t *test, * indicates p < 0.05; ^a ^250 μg/Kg.

**Table 2:** Serum and CSF TNF_α _after ICV administration of LPS in rats

	Control	LPS^b^	p
Serum (30 min)	0.7 ± 0.3	121.6 ± 40.0	0.001*
Serum (24 h)	1.1 ± 0.3	1.0 ± 0.3	0.945
CSF (30 min)	38.8 ± 9.8	75.1 ± 24.9	0.215
CSF (24 h)	1.7 ± 1.2	19.6 ± 4.5	0.006*

Values are mean (pg/mL) ± standard error (n = 5). Statistical analysis was performed using Student's *t *test, * indicates p < 0.05; ^b ^25 μg

## Discussion

S100B has been proposed as a marker of brain injury and its elevation in CSF has been interpreted as a signal of astroglial activation [10,11]. Moreover, it has been assumed that S100B from CSF easily crosses the blood brain barrier and that a S100B increment in peripheral blood is indicative of brain injury. However, in some pathophysiological conditions other interpretations are possible and, consequently, an intense debate has been developed, mainly because there are extra-cerebral sources of S100B [31].

Serum levels of S100B after exposure to LPS have been measured in some studies. S100B protein blood levels in fetal sheep were found to be significantly higher 1 h after LPS administration (intravenous [IV], 5 mg/Kg) and to return to baseline between 12 and 72 h after exposure [32]. Similarly, in Sprague-Dawley rats, this quantity of LPS is able to induce an increase in serum S100B 5h later [33]. In our study, ICV (2.5 ng) or IP. administration (0.25 mg/Kg) of LPS to Wistar rats did not alter serum S100B levels, measured 30 min and 24 h after exposure. This discrepancy could be due to the different quantities of LPS employed, to its method of administration, or to the type of animal. Importantly, LPS (IV 2 ng/Kg), when given to humans, is not able to induce significant changes in serum S100B at 1 h or 8 h post treatment [34].

In addition to measuring serum S100B, we also evaluated S100B levels in CSF, astrocyte cultures and acute hippocampal slices of rats exposed to LPS. Astrocytes are thought of as active cells in the immune response, because they have receptors for this response (e.g TLR4) and are able to secrete cytokines [19,35]. We found an increase in CSF S100B after LPS both for ICV (early and persistent response) and for IP administration (early and transient response). Notice that LPS is potentially able to cross the blood-brain barrier [36]. Clearly no immediate increment in serum S100B occurred in either condition. This suggests brain-specific, LPS-induced release of S100B, i.e., peripheral immune cells stimulated by LPS did not release or cause a detectable S100B release from potential extra-cerebral sources of S100B (e.g. adipocytes). In other words, these data suggest different LPS-sensitivities for S100B secretion in central and peripheral S100B-expressing cells. Conversely, we observed an immediate serum TNFα increase after LPS administration by both ICV and IP routes. It has been proposed that TNFα is able to mediate S100B secretion in astrocytes [37]. However, under LPS stimulation, our results regarding the profiles of increases in TNFα and S100B in serum and CSF suggest independent responses (Table 3).

**Table 3:** Qualitative comparison of TNFα and S100B levels in serum and CSF after LPS administration

		TNFα	S100B
LPS IP	Serum (30 min)	↑	--
	Serum (24 h)	↑	--
	CSF (30 min)	--	↑
	CSF (24 h)	--	--

LPS ICV	Serum (30 min)	↑	--
	Serum (24 h)	--	--
	CSF (30 min)	--	↑
	CSF (24 h)	↑	↑

↑ indicates a significant increase compared to control, with a significance level of p < 0.05; -- indicates no significant difference compared to control. See Table 1, Table 2 and Figure 1 for details.

Other aspects must be emphasized. The increase in CSF S100B levels that we found was not accompanied or followed by an increase in serum S100B levels, at least in measurements made at the evaluated times (30 min and 24 h after LPS). This increase in CSF S100B was rapid (i.e. detected in 15 min) and lasting (for at least 24 h). Notice that control animals for the experiments involving ICV administration of LPS exhibited higher levels of CSF S100B (Figure 1A) than did controls for IP administration (Figure 1C), suggesting a response to the invasive procedure.

Astrocytes in culture secreted S100B directly in response to LPS (from 10 μg/mL upward) at 1h, but at 24 h a decrease in secretion (dependent on LPS concentration) was observed even at lower concentrations. This suggests a biphasic response, i.e. an increase in S100B secretion, followed by a decrease. This profile has been observed in astrocyte cultures under other conditions, such as exposure to beta-hydroxybutyrate [38]. This rapid and transient stimulation of S100B secretion in astrocyte cultures was also observed for the cytokine IL-1β, but without a decrease at 24 h [12]. This finding could suggest that the LPS effect is direct and independent of secondarily-released IL-1β. Other studies have reported an increase in cell content of S100B in C6 glioma cells after 24 h of exposure to IL-1β [39] or no change in astrocyte cultures after 48 h [40] and a decrease in S100B content in cultured astrocytes after 3 days of exposure to TNFα [37]. However, these studies did not measure S100B secretion adequately and it is not possible to speculate about a secondary effect of these two cytokines on S100B secretion after long-term LPS exposure under the conditions used here. Therefore, in agreement with our working hypothesis, it appears that LPS is able to directly modulate S100B secretion.

In addition, when we used acute hippocampal slices to evaluate S100B secretion at 1 h, we also observed an increase in S100B secretion with LPS at 10 μg/mL, but conversely we observed a decrease in LPS at 0.1 or 1 μg/mL. These preparations are complex from a cellular view, i.e. in addition to astrocytes, they contain active microglia and neurons, which makes interpretation of the control of S100B release difficult. However, a similar result, obtained in response to endothelin-1, has also been observed [24]. This compound, due to its blocking effect on gap junctions, increases S100B secretion in astrocyte cultures in the first hour, but after 6 hours decreases S100B secretion. Similarly, in acute hippocampal slices, endothelin-1 decreases S100B secretion at 1 h. Potentially, both LPS and endothelin-1 down-regulate gap junction proteins. Although we have no doubt about the effects of LPS and endothelin-1 on S100B secretion in acute hippocampal slices, we have no explanation for this effect, when compared to that observed in isolated astrocytes, at this moment.

Secreted S100B is a very small part of total cell content (less than 0.5% is found in the medium of astrocyte cultures at 24 h) and changes in S100B secretion are not necessarily accompanied by changes in the cell content [31]. In fact, in our experiments LPS changed S100B secretion without affecting cell content of this protein. On the other hand, GFAP content was increased by all concentrations of LPS used, indicating astroglial activation. This is in agreement with previous reports about the effects of LPS on astrocyte cultures [22,23]. This reinforces the idea that GFAP and S100B have distinct regulatory mechanisms of expression and that astrogliosis (as assessed by GFAP increment) can either be accompanied or not accompanied by changes in cell S100B content [41].

Another interesting aspect of our findings is decreased glutathione content after LPS exposure. The decrease in glutathione content in astrocytes at 24 h (but not at 1 h) is possibly associated with up-regulation of glutathione-S-transferase, as observed very recently [22]. Part of the decrease could involve an intense exportation of this peptide, since it serves as an extracellular antioxidant, and also provides substrates for neuronal synthesis of glutathione [42]. In addition, we also found a decrease in glutathione content in acute hippocampal slices exposed to LPS.

In spite of this decrease in antioxidant defense, both preparations exhibited excellent viability and integrity, based on MTT reduction assays, neutral red incorporation and LDH release. These assays, performed in parallel to assays for S100B measurements, allowed us to be emphatic throughout the text about S100B *secretion*, instead of S100B *release*.

Although S100B has cytokine-like actions (e.g. [43]), some caution should be taken in the categorization of S100B as a cytokine. In contrast to classical cytokines, S100B is not produced exclusively for secretion; only a very small part is exported. More recently, some authors have suggested that S100B, like other members of the S100 family, may act as an alarmin or damage-associated molecular pattern (see [44] for a review). However, independently of these conceptions, our data suggest that S100B secretion is modulated by LPS. In fact, secretion of S100B might be protective during the initial phase of LPS challenge. In contrast, prolonged LPS treatment results in a dose-dependent decrease in S100B secretion from astrocytes. This indicates that one potential effect of long-lasting exposure to LPS might be decreased secretion of trophic factors from astrocytes.

It should be noted that some aspects of the effect of LPS remain unclear. Firstly, is the effect of LPS mediated exclusively by TLR-4 in astrocytes? We cannot rule out other possibilities at this moment, since LPS could be acting on other receptors (e.g. CD14 and LBP [45]. Secondly, it is still not clear whether LPS can affect S100B secretion in other S100-expressing cells. There are many extracerebral S100B-expressing cells that affect serum S100B levels [46] and these, apparently, were not mobilized under our conditions of LPS stimulation. However, further studies must investigate specific extracerebral sources of S100B. For example, it is known that enteroglia respond to LPS by increasing levels of S100B mRNA [47]. Third, whether gram-negative infectious agents could cause similar effects on S100B secretion, mediated by LPS release, is not clear at the moment. Interestingly, serum S100B was found to be increased in patients with cerebral and extracerebral infectious disease [48]. In that study, S100B elevation was generally higher in patients with cerebral infections than in extracerebral infections. However, specific and chronic effects of gram-negative bacteria on central and peripheral S100B deserve further investigation.

## Conclusions

Our data suggest that S100B secretion in brain tissue is stimulated rapidly and persistently (at least for 24 h) by ICV administration of LPS. Moreover, no changes were observed in serum levels of this protein. This profile is quite different from that of TNFα, a canonical inflammatory cytokine. In isolated astrocytes and acute hippocampal slices, we observed a direct stimulation of S100B secretion by LPS at a concentration of 10 μg/mL, mediated by TLR4. However, in astrocyte cultures, lower levels of LPS were able to induce a decrease in S100B secretion 24 h afterwards, without significant changes in the intracellular content of S100B. In addition, after 24 h of exposure of astrocytes to LPS, we observed a decrease in glutathione and an increase in GFAP. Together, these data contribute to our understanding of the effect of LPS on astrocytes, particularly on S100B secretion, and help us to interpret cerebrospinal fluid and serum changes of this protein in neuroinflammatory diseases and brain disorders in general. Moreover, S100B-expressing tissues may be differentially regulated, since LPS did not lead to increases in serum S100B.

## Competing interests

The authors declare that they have no competing interests.

## Authors' contributions

Conception and design of experiments: MCG, LST, MCL and CAG

Acquisition, analysis and interpretation of data: MCG, LST, MCL, FG, CDR, EN, DSE and LR

Writing and/or critical review of article: MCG, LST, MCL and CAG

All authors have read and approved the final version of the manuscript.
